# Multimodality Cardiac Imaging in Young and Veteran Athletes: Updates on Atrial Function Assessment, Arrhythmia Predisposition and Pathology Discrimination

**DOI:** 10.3390/jcm12030797

**Published:** 2023-01-19

**Authors:** Emmanuel Androulakis, Francesco Perone

**Affiliations:** 1 Sports Cardiology & Inherited Cardiac Conditions Department, St George’s Hospital NHS Foundation Trust, London SW17 0QT, UK; 2 Cardiac Rehabilitation Unit, Rehabilitation Clinic “Villa delle Magnolie”, Castel Morrone, 81020 Caserta, Italy

Sports physicians and physiologists have aimed to assess exercise in young and master athletes so as to work out their conditioning levels and design training programs accordingly. Cardiopulmonary exercise testing (CPET) provides a thorough assessment of the physiologic responses of the pulmonary, cardiovascular, muscular, and cellular oxidative systems to exercise in both athletic individuals and those with cardiovascular conditions, and has been used successfully to assess post-COVID-19 infection sequelae in competitive athletes [1,2,3].

On the other hand, athletic training is known to induce morphological and functional cardiovascular adaptations of both ventricles and atria, collectively known as athlete’s heart. Apart from advanced echocardiographic methods, cardiac magnetic resonance (CMR) is an imaging modality with a high spatial resolution and no exposure to ionizing radiation, and has the benefit of tissue characterization by exploiting gadolinium-based contrast late enhancement as a marker of myocardial fibrosis. Its additional novel developments in tissue characterization, particularly in the last 5–10 years, have been increasingly used in clinical practice in pathology discrimination, particularly with inherited cardiomyopathy. These may provide an accurate diagnosis in challenging cases and may differentiate from various pathologies, but, at the same time, provide insights into atrial structure, function, and future risk of atrial and ventricular arrhythmia [4]. 

More specifically, biatrial function was estimated by peak atrial longitudinal and contraction strains, before and after races in master athletes. The results of a cross-sectional study by the group of D’Ascenzi in Italy did not confirm the hypothesis of acute atrial dysfunction induced by ultra-endurance exercise, even though one athlete developed atrial fibrillation during the race [5]. Furthermore, observational data from Zürich, based on patients with definite arrhythmogenic right ventricular cardiomyopathy (ARVC) compared with athletes, showed that ARVC patients present with a significantly larger right atrial volume index (RAVI) compared to athletes, resulting in a greater RAVI/LAVI ratio. Based on an ROC analysis, a score was developed that included the following parameters: indexed right/left atrial volume ratio (RAVI/LAVI ratio), NT-proBNP, RV outflow tract measurements, tricuspid annular motion, precordial T-wave inversion, and depolarization abnormalities according to 2010 Task Force Criteria. A score of 6/12 points yielded a specificity of 91% and an improved sensitivity of 67% for ARVC diagnosis as compared to a sensitivity of 41% for the Task Force Criteria [6].

Moreover, in a single-center study, again from Switzerland, 12-lead ECG, signal-averaged ECG, 24 h Holter ECG, and advanced left atrial echocardiographic data were prospectively collected in patients with hypertrophic cardiomyopathy, hypertensive disease, and endurance athletes. Structural and electrical atrial remodeling is more advanced in hypertrophic cardiomyopathy compared to hypertensive disease and endurance athletes [7]. Another recent advance which might play an important role in imaging diagnostics is HyperDoppler. It is a new echocardiographic color Doppler-based technique that can assess intracardiac flow dynamics. A feasibility and reproducibility study of this technique was recently performed on unselected patients, and its capability to differentiate measures of vortex flow within the left ventricle (LV) in normal sedentary subjects, athletes, and patients with heart failure was assessed. It was shown that HyperDoppler is a feasible, reliable, and practical technique for the assessment of LV flow dynamics, and may distinguish between normal subjects and patients with heart failure [8].

Lastly, and quite importantly, the Italian group from Padova by Corrado et al. has summarized important recent work on the diagnostic assessment of athletes with premature ventricular beats (PVBs) and contrast-enhanced CMR imaging. The morphology, complexity, and exercise inducibility of PVBs can help estimate the probability of an underlying heart disease. Based on these features, the CMR imaging and assessment of non-ischemic scars, as well as their patterns and extent, may be indicated even when echocardiography is normal [9]. 
